# Massive Biochemically Silent Pheochromocytoma Masquerading as Nonfunctioning Adrenocortical Cancer

**DOI:** 10.1210/jcemcr/luad061

**Published:** 2023-06-07

**Authors:** Shejil Kumar, Katherine Wu, Natassia Rodrigo, Anthony Glover

**Affiliations:** Department of Diabetes, Endocrinology & Metabolism, Royal North Shore Hospital, Sydney, NSW 2065, Australia; Department of Diabetes, Endocrinology & Metabolism, Royal North Shore Hospital, Sydney, NSW 2065, Australia; Department of Diabetes, Endocrinology & Metabolism, Royal North Shore Hospital, Sydney, NSW 2065, Australia; Northern Sydney Clinical School, Faculty of Medicine, University of Sydney, Sydney, NSW 2065, Australia; Endocrine Surgical Unit, Royal North Shore Hospital & Northern Clinical School, Sydney, Medical School, Faculty of Medicine and Health, University of Sydney, Sydney, NSW 2065, Australia; The Kinghorn Cancer Centre, Garvan Institute of Medical Research, St. Vincent's Clinical School, Faculty of Medicine, University of New South Wales, Darlinghurst, NSW 2010, Australia

**Keywords:** pheochromocytoma, adrenal gland, biochemically silent, non-functioning, normetanephrines, metanephrines

## Abstract

Pheochromocytomas are rare catecholamine-secreting neuroendocrine tumors of the adrenal medulla chromaffin cells, usually associated with features of catecholamine excess. Clinically and biochemically silent pheochromocytoma without adrenergic symptoms or elevated catecholamine concentrations are rare. A 71-year-old female presented with acute right flank pain with abdominal computed tomography (CT) scan revealing a hemorrhagic right adrenal mass. She had no preceding adrenergic symptoms, and normal serum electrolytes, on a background of well-controlled hypertension on amlodipine monotherapy. After conservative management and discharge, an outpatient CT adrenal scan confirmed an 88 × 64 mm right adrenal mass demonstrating intense avidity (maximum standardized uptake value, 20.2) on fluorodeoxyglucose F 18-positron emission tomography (FDG-PET)/CT scan. Biochemical screening supported a nonfunctional adrenal lesion with normal-range plasma normetanephrines and metanephrines. She underwent a right adrenalectomy for presumed nonfunctioning adrenocortical cancer; however, histopathology demonstrated a 120-mm pheochromocytoma. Succinate dehydrogenase subunit B (SDHB) and fumarate hydratase (FH) staining were retained; however, weakly positive 2SC staining raised concerns for FH-deficient pheochromocytoma. Germline DNA sequencing was negative for pathogenic RET, VHL, SDHB, SDHD, or FH variants. Tumor cells stained positive for tyrosine hydroxylase and negative for dopamine β hydroxylase. Four months postoperatively, progress FDG-PET/CT scan demonstrated no focal avidity. Massive biochemically silent pheochromocytomas are exceedingly rare, and we discuss various mechanisms that may predispose patients to this phenomenon.

## Introduction

Pheochromocytomas are rare neuroendocrine tumors arising from chromaffin cells of the adrenal medulla [1]. Typically, pheochromocytomas secrete excess catecholamines (epinephrine and/or norepinephrine), and patients often exhibit features of catecholamine excess such as headaches, sweats, palpitations, and hypertension. Approximately 5% of adrenal incidentalomas are considered pheochromocytomas, and so pheochromocytomas with minimal or no adrenergic symptoms are not uncommon [1]. However, a “biochemically silent” pheochromocytoma without evidence of excess circulating concentrations of catecholamines or catecholamine metabolites is exceedingly rare [1]. We present a rare case of a massive, biochemically silent pheochromocytoma. The tumor tissue demonstrated extensive central hemorrhagic, cystic, and necrotic changes, whereas immunohistochemistry was negative for dopamine β hydroxylase, raising several hypotheses for how such an unusual phenomenon may occur.

## Case Presentation

A 71-year-old female presented to her local emergency department in May 2022 with severe acute right flank pain. She was hemodynamically stable (blood pressure, 103/66 mm Hg; heart rate, 72 beats/min). An urgent computed tomography (CT) scan of her chest, abdomen, and pelvis revealed right-sided subsegmental pulmonary emboli and a 110 × 100-mm hemorrhagic right-sided adrenal mass with mass effect on the adjacent kidney and inferior vena cava (IVC). She had no preceding adrenergic symptoms to suggest pheochromocytoma or overt signs of Cushing syndrome. She had well-controlled hypertension on amlodipine monotherapy with no history of postural dizziness or hypotension. She had no history of malignancy, and a recent colonoscopy and mammogram were unremarkable. There was no family history of adrenal lesions or multiple endocrine neoplasia syndromes. Serum electrolytes were unremarkable. CT mesenteric angiogram scans did not demonstrate active arterial extravasation. She was managed conservatively with analgesia and prophylactic anticoagulation and discharged from hospital 7 days later for further workup.

## Diagnostic Assessment

**Figure 1.**
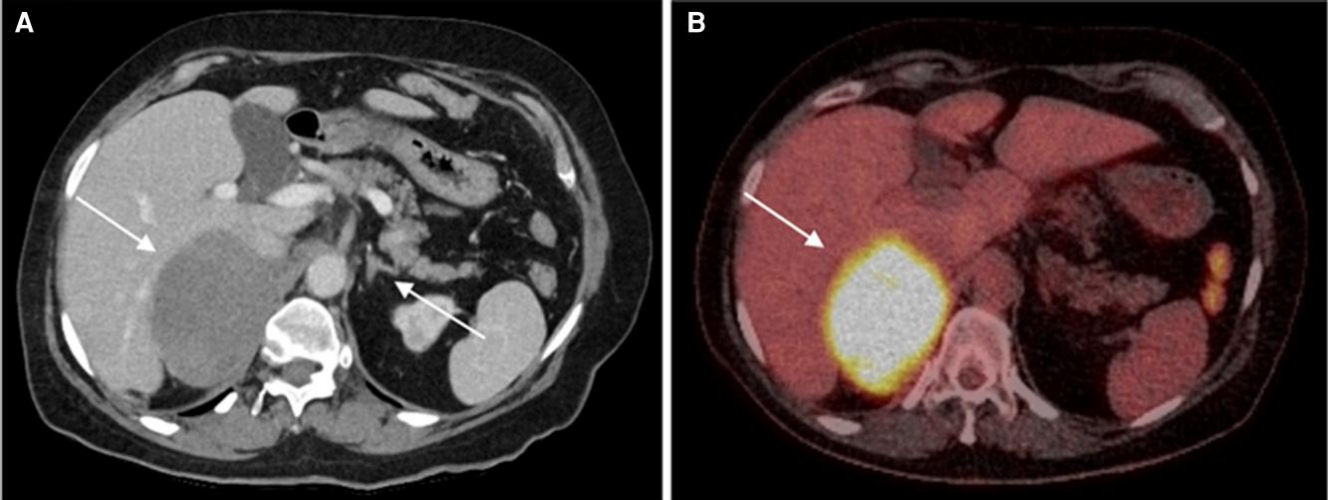
Outpatient structural and functional adrenal imaging. Axial-view abdominal CT scan demonstrates an 88 × 64-mm homogeneous hypodense right adrenal mass with normal-appearing left adrenal gland (A). Axial-view FDG-PET/CT scan demonstrates diffusely increased avidity (SUVmax, 20.2) of the right adrenal mass (B).

On outpatient follow-up, biochemical screening, performed 6 weeks after the initial presentation, was negative for a functioning adrenal lesion, including plasma metanephrines and normetanephrines, serum aldosterone/renin ratio, and late-night salivary cortisol (Table 1). Follow-up adrenal CT scan confirmed an 88 × 64-mm homogeneous right adrenal mass with normal left adrenal gland (Fig. 1A). Because of the size of the mass, an fluorodeoxyglucose F 18-positron emission tomography (FDG-PET)/CT scan was performed that showed an intensely avid (maximum standardized uptake value, 20.2) right adrenal mass with no other abnormal focal avidity (Fig. 1B). Open surgical resection was recommended following multidisciplinary team discussion because of a risk of malignancy.

**Table 1.** Adrenal functional test results negative for functioning adrenal tumor

Parameter	Result	Reference range
**Blood**		
Plasma normetanephrine	430 (0.43)	<1280 pmol/L (<1.28 nmol/L)
Plasma metanephrine	90 (0.09)	<447 pmol/L (<0.45 nmol/L)
Plasma 3-methoxytyramine	110 (0.11)	<181 pmol/L (<0.18 nmol/L)
Aldosterone (upright)	229 (8.26)	100–950 pmol/L (3.6–34.3 ng/dL)
Renin (upright)	11 (24.2)	3.3–41 mIU/L (7.3–90.2 pg/mL)
Aldosterone/renin ratio	21	<70
**Saliva**		
Midnight cortisol	3 (0.11)	<8 nmol/L (<0.29 mcg/dL)

## Treatment

She was readmitted in July 2022 for elective right adrenalectomy for presumed nonfunctioning adrenocortical cancer. The tumor was adherent to the IVC, the liver capsule, and diaphragm, requiring open adrenalectomy and partial resection and repair of these structures. She experienced intra- and postoperative hypotension requiring metaraminol infusion overnight in the intensive care unit. She was commenced empirically on IV hydrocortisone 50 mg 8-hourly because of initial concerns of a cortisol-secreting adrenocortical cancer. However, prehydrocortisone cortisol levels were normal (484 nmol/L, 17.5 mcg/dL); therefore, hydrocortisone was ceased.

## Outcome and Follow-up

**Figure 2.**
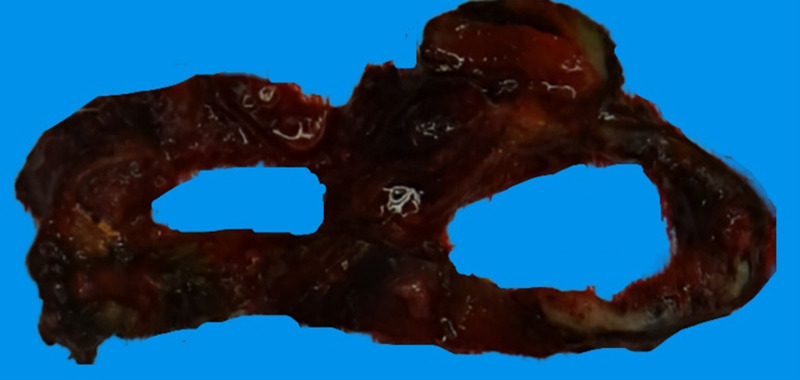
Macroscopic cystic appearance of right adrenal mass.

**Figure 3.**
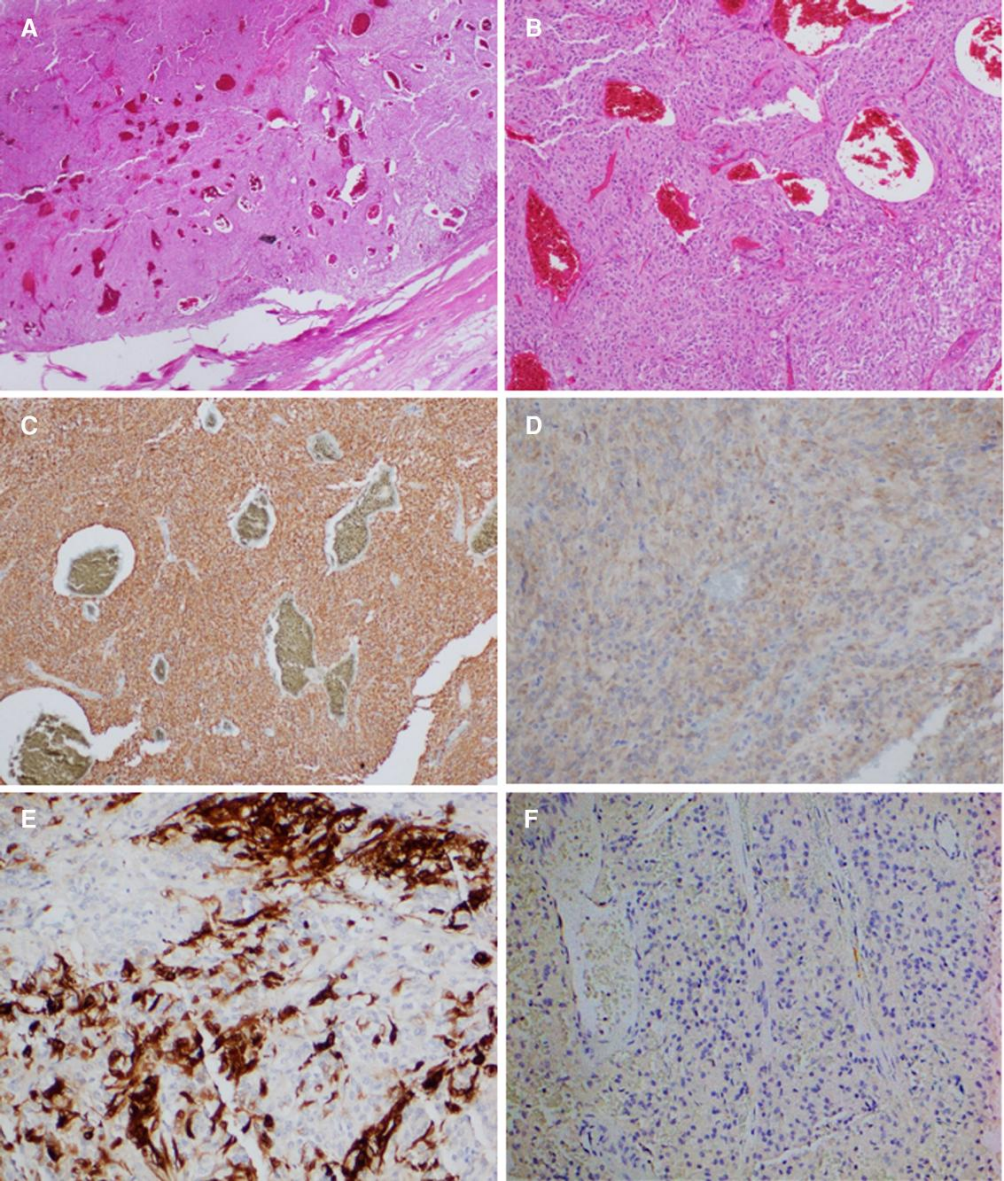
Microscopic features of pheochromocytoma tumor cells. Hematoxylin and eosin-stained tissue demonstrates clusters of tumor cells with basophilic cytoplasm and uniform nuclei and central hemorrhagic and necrotic changes with dilated blood-filled sinusoids (A: 4× power; B: 10× power). Immunohistochemistry demonstrated strongly positive normal pattern of staining for fumarate hydratase (C) and weakly positive abnormal pattern of staining for 2SC (D), raising suspicion for fumarate-hydratase–deficient pheochromocytoma. Tumor cells stained positive for tyrosine hydroxylase (E) and negative for dopamine beta hydroxylase (F). Images courtesy of Dr Mahsa Ahadi, Department of Anatomical Pathology, Royal North Shore Hospital.

Macroscopically, the right adrenal mass appeared cystic (Fig. 2). Histopathology surprisingly demonstrated a 120-mm pheochromocytoma. Tumor cells had eosinophilic to amphophilic cytoplasm with relatively uniform nuclei containing speckled chromatin, which is classic for pheochromocytoma. There was extensive central hemorrhage and necrosis (Fig. 3A and 3B). The tumor was noted to have invaded surrounding soft tissue and the IVC with an R-0 resection. Tumor staining for succinate dehydrogenase subunit B (SDHB) and fumarate hydratase (FH) were retained (normal pattern), however, was positive for 2SC (abnormal) (Table 2, Fig. 3C and 3D), raising suspicion for an FH-deficient pheochromocytoma. Ki-67 index was 5%. Tumor cells showed positive staining for tyrosine hydroxylase but negative staining for dopamine β hydroxylase (DBH) (Fig. 3E and 3F). DBH immunohistochemistry was repeated on a separate case of secretory pheochromocytoma and another patient with normal adrenal medullary tissue, with both being positive.

**Table 2.** Immunohistochemistry results of tumor cells

Parameter	Result
Synaptophysin	Positive
Chromogranin-A	Positive
SF1	Negative
Melan-A	Negative
Ki-67	5% (hot spots)
SDHA	Intact
SDHB	Intact
Fumarate hydratase	Positive, strong
2SC	Weak cytoplasmic staining (positive/abnormal)
Tyrosine hydroxylase	Positive
Dopamine β hydroxylase	Negative

SDHA, succinate dehydrogenase subunit A; SDHB, succinate dehydrogenase subunit B; SF1, steroidogenic factor 1.

Subsequent germline DNA sequencing was negative for pathogenic variants in RET, VHL, SDHB, SDHD, and FH genes (DBH was not analyzed). Repeat FDG-PET/CT scan in November 2022 demonstrated no residual, nodal, or distant metastatic disease. Following multidisciplinary team discussion, she underwent adjuvant radiotherapy to the right adrenal bed in December 2022 (50 Gy over 25 fractions).

## Discussion

Clinically and biochemically silent pheochromocytomas are rare. The term “clinically silent” has been used in the literature to describe cases in which patients present without the classical signs or symptoms associated with catecholamine excess such as sweating, headaches, palpitations, and/or hypertension [2]. The clinical presentation of pheochromocytoma is diverse and dependent on synthesis and release of catecholamines into circulation, size of the tumor, associated mass effect, and whether there is associated hemorrhage [2]. Indeed, our patient's pheochromocytoma was only discovered in the context of acute flank pain secondary to adrenal hemorrhage. In all patients with spontaneous adrenal hemorrhage, an underlying adrenal tumor should be considered. In a systematic review of 133 cases of adrenal hemorrhage with underlying adrenal tumor, 50% of tumors were pheochromocytomas (median tumor size, 7 cm), likely related to rapid tumor growth leading to increased intracapsular pressure and predisposing to capsular tear in an already highly vascular tumor [3]. Other neoplastic causes include adrenal metastasis (14%, most often bilateral), primary adrenocortical cancer (7%), and adrenal adenoma (4%). In acute adrenal hemorrhage, clinical assessment can be challenging because of nonspecific symptoms and difficulty interpreting serum cortisol and catecholamine concentrations during physiological stress. Further, acute adrenal hemorrhage manifests radiographically as adrenal swelling displaying an oval-round appearance with high density and can mask an underlying neoplasm and make delineation of benign and malignant lesions difficult [3, 4]. Hence, hormonal workup should be conducted in the outpatient setting when the patient's condition has stabilized, and imaging repeated within 3 months [4].

“Biochemically silent” or “nonsecretory” are terms used to describe tumors that do not result in excess circulating concentrations of catecholamines and their metabolites in sampling of blood and/or urine [2]. The causes for this are uncertain and may be related to clinician/diagnostic factors (eg, incorrect screening test, sampling errors, tumor factors such as size, extent of catecholamine secretion and presence of hemorrhagic, cystic, and/or necrotic changes). Other factors that may contribute to absence of signs and symptoms in patients with pheochromocytomas include the types of catecholamines secreted and pattern of secretion (eg, episodic, persistent).

The main explanation for clinically silent pheochromocytoma is discovery at an early stage. Advancements in abdominal and whole-body imaging and increased uptake of surveillance in patients at risk of pheochromocytoma because of hereditary predisposition or previous tumor have led to increased detection of clinically and/or biochemically silent pheochromocytomas [4]. These incidentalomas are typically smaller than 3 cm and often too small to be able to secrete sufficient catecholamines to produce physical and laboratory manifestations of pheochromocytoma [1]. Eisenhofer et al demonstrated prospectively that in the majority of cases of pheochromocytoma, concentrations of plasma catecholamine metabolites can predict tumor diameter, whereby tumor size positively correlates with degree of elevation of plasma catecholamine metabolite concentrations [5].

It follows that massive pheochromocytoma, such as in our case, rarely remains biochemically silent and, if so, must be explained by factors other than tumor size. A recent systematic review identified 157 cases reported with “silent” or “nonfunctioning” pheochromocytoma and paraganglioma, of which 38% were adrenal tumors, and the most common reason for biochemical screening was detection of an incidental adrenal lesion (62%) because of abdominal or other nonspecific symptoms [2]. However, only 40 patients and 43 patients, respectively, had plasma and urinary metanephrines in the normal range, and the majority of cases defined by authors as “biochemically silent” did not report results or reference intervals for plasma and/or urinary catecholamine metabolites. This reinforces the rarity of our case as truly “biochemically silent,” despite the presence of a massive pheochromocytoma. The average maximal tumor diameter was 55 mm across 81 cases, less than half the size of the 120-mm tumor detected in our patient [2]. The mechanisms whereby massive pheochromocytoma can be biochemically silent are uncertain, although it has been postulated that, as the tumor enlarges, it may exceed its blood supply, resulting in necrosis and hemorrhage within the tumor and ultimately cystic changes as the liquefied contents are resorbed, such as in our case [6]. In 29 cases of biochemically silent pheochromocytomas reporting tumor characteristics in the previously mentioned systematic review, 31% had cystic changes and 38% had evidence of hemorrhage and/or necrosis [2]. It has been postulated that extensive necrosis and hemorrhagic and cystic changes may cause significantly decreased catecholamine-producing cells in pheochromocytoma tissue [7]; this may have contributed to nonsecretory pheochromocytoma in our case.

Another mechanism for clinically silent pheochromocytoma is the degree of intratumoral metabolism of catecholamines. Catecholamines are secreted from chromaffin cells or pheochromocytoma tumor cells by exocytosis and leak continuously from storage vesicles into the cytoplasm, where catechol-O-methyltransferase metabolizes norepinephrine and epinephrine to inactive metabolites normetanephrine and metanephrines, respectively, which then diffuse passively into the circulation [2, 8]. This phenomenon may explain why some large tumors with high concentrations of circulating catecholamine metabolites are clinically silent but does not explain the concept of biochemically silent disease.

Tyrosine hydroxylase is the enzyme that converts tyrosine to levodopa, whereas DBH converts dopamine to norepinephrine in the catecholamine synthesis pathway. Hence, lack of expression of tyrosine hydroxylase and/or DBH in pheochromocytoma tumor cells, as demonstrated by immunohistochemistry in our patient, may result in a defect in catecholamine synthesis independent of tumor size [2]. Dopamine has minimal action on α and β adrenoreceptors [2, 9] and thus lack of DBH, which results in accumulation of dopamine as opposed to norepinephrine/epinephrine can predispose to clinically and biochemically silent pheochromocytoma. DBH deficiency in tumor cells (and hence impaired conversion from dopamine to norepinephrine) may have predisposed to elevated circulating dopamine levels [9] that were not directly measured in our patient; however, plasma 3-methoxytyramine concentration was within the normal range, making a diagnosis of dopamine-secreting pheochromocytoma unlikely [2].

Additionally, it is important to consider other explanations for apparently biochemically silent pheochromocytoma, including false-negative screening. Approximately half of all biochemically functioning pheochromocytomas secrete excess norepinephrine, whereas the remainder secrete a mixture of norepinephrine and epinephrine. Although these catecholamines are directly secreted by pheochromocytomas, measuring only plasma and urinary epinephrine/norepinephrine levels for diagnosis is unfavorable given their episodic release into the circulation. Rather, the clinical practice guideline recommendations from the Endocrine Society for biochemical diagnosis of pheochromocytoma is to measure circulating levels of metanephrines and normetanephrines [10], the O-methylated metabolites of catecholamines that are continuously produced within chromaffin cells [1], with plasma concentrations generally preferred because of higher sensitivity [2]. Despite this, in Constantinescu et al's systematic review, only 45 cases had plasma metanephrines reported and 57 cases had urinary metanephrines reported [2].

Pheochromocytoma is rare but still warrants consideration in patients with adrenal masses with negative biochemical results and nonspecific symptoms. Massive biochemically silent pheochromocytoma is exceedingly rare, particularly because tumor size tends to correlate with concentration of circulating catecholamine metabolites in pheochromocytoma. Potential mechanisms demonstrated in our case include lack of tumor expression of catecholamine-synthesizing enzymatic machinery (eg, DBH) and hemorrhagic, cystic, and/or necrotic changes potentially related to large tumor size.

## Learning Points

- Pheochromocytomas represent the majority of adrenal tumors associated with spontaneous adrenal hemorrhage.
- Pheochromocytomas should be considered in the differential diagnosis for an adrenal mass despite absence of adrenergic symptoms and elevated circulating catecholamine metabolites.
- Biochemically silent pheochromocytoma is rare, particularly in cases of massive pheochromocytoma.
- Factors that may contribute to biochemically silent pheochromocytoma include false-negative biochemical screening, absence in tumor tissue of necessary enzymes for catecholamine synthesis (eg, tyrosine hydroxylase, dopamine beta hydroxylase), and cystic, hemorrhagic, and/or necrotic changes.

## Data Availability

Data sharing is not applicable to this article as no datasets were generated or analyzed during the current study.

## Abbreviations

CT: computed tomography
DBH: dopamine β hydroxylase
FH: fumarate hydratase
IVC: inferior vena cava
SDHB: succinate dehydrogenase subunit B
